# Gait impairments in patients with bilateral vestibulopathy and chronic unilateral vestibulopathy

**DOI:** 10.3389/fneur.2025.1547444

**Published:** 2025-02-27

**Authors:** Anissa Boutabla, Rebecca Revol, Marys Franco Carvalho, Gautier Grouvel, Julie Corre, Jean-François Cugnot, Samuel Cavuscens, Maurizio Ranieri, Meichan Zhu, Christopher McCrum, Raymond van de Berg, Stéphane Armand, Angélica Pérez Fornos, Nils Guinand

**Affiliations:** ^1^Division of Otorhinolaryngology Head and Neck Surgery, Geneva University Hospitals and University of Geneva, Geneva, Switzerland; ^2^Kinesiology Laboratory, Geneva University Hospitals and University of Geneva, Geneva, Switzerland; ^3^Division of Clinical Neurosciences, Geneva University Hospitals, Geneva, Switzerland; ^4^Department of Nutrition and Movement Sciences, NUTRIM School of Nutrition and Translational Research in Metabolism, Maastricht University Medical Center+, Maastricht, Netherlands; ^5^Division of Balance Disorders, Department of Otorhinolaryngology and Head and Neck Surgery, Maastricht University Medical Center+, Maastricht, Netherlands

**Keywords:** vestibular function, motion analysis, bilateral vestibulopathy (BV), unilateral vestibulopathy, gait

## Abstract

Vestibular deficits often lead to unsteady gait, affecting quality of life and increasing fall risk. This study aimed to identify gait impairments in chronic vestibulopathy. Ten patients with bilateral vestibulopathy (BV), 10 patients with chronic unilateral vestibulopathy (UV), and 10 healthy participants (HS) participated. Spatio-temporal parameters were computed during walking at various self-selected walking speeds (slow, comfortable, and fast) using motion capture system with additional assessment usingclinical gait tests [functional gait assessment (FGA), tandem walk (TW), Timed Up and Go test (TUG)], and symptom severity [Dizziness Handicap Inventory (DHI)] were assessed and compared between the three groups. BV and UV patients showed significantly slower walking speeds, shorter step lengths, and broader step widths compared to HS, but similar cadence. Significant differences were also seen in stance phase, double and single support phases at comfortable and slow speeds, but not at fast speed. BV patients, but not UV patients, had worse FGA scores than HS, reflecting their reported difficulties in specific tasks requiring greater postural control. Tandem walk performance was lower in BV patients compared to the other groups, whereas there was no significant differences in TUG scores. Cluster analysis revealed two distinct clusters: one with all HS and most UV patients (70%), and another with most BV patients and 30% of UV. Overall, this study highlights how altered vestibular function impacts gait outcomes. These findings can aid clinicians in evaluating gait in patients with vestibular deficits and monitoring rehabilitation interventions.

## Introduction

By sensing head accelerations, the peripheral vestibular system plays a key role in the multisensory balance system. Impairment of peripheral vestibular function can lead to abnormal gait patterns and gait unsteadiness (1). Heterogenous etiologies lead to impaired the vestibular function, although in up to 50% of the cases no clear cause is found (2, 3). The onset is either abrupt (i.e., acute unilateral vestibulopathy) or develop slowly (i.e., presbyvestibulopathy or vestibular schwannoma). It can also result from successive acute episodes (i.e., Menière Disease). Vestibulopathy can be complete or partial, it can affect one [unilateral vestibulopathy (UV)] or both ears [bilateral vestibulopathy (BV)]. All BV patients report a certain degree of imbalance (i.e., gait unsteadiness) (4, 5). For UV patients, central compensation may decrease the impact of the unilateral vestibular loss. Additionally, adequate vestibular rehabilitation can efficiently enhance the benefits of central compensation (6–9). Nevertheless, it has been estimated that up to one third of UV patients remain symptomatic in the long term. Within this subset of UV patients, chronic dizziness is the most frequent complaint with a prevalence of 98% (6, 10). Gait unsteadiness in dynamic conditions is the second most frequent complaint, with a prevalence of 81% (4, 6). Both for BV and UV patients, gait unsteadiness tends to worsen when moving in low-light settings or on uneven ground (11). Moreover, a majority of BV patients and a smaller proportion of UV patients report experiencing oscillopsia (blurred vision) during dynamic activities such as walking, as well as more subtle symptoms related to cognition and emotions (12). Consequently, both BV and UV can significantly impede the patient’s ability to carry out normal daily activities and quality of life can be significantly affected (11–14).

Although numerous tools and protocols exist to quantify gait and evaluate associated symptoms, they are not systematically integrated into diagnostic and care protocols for vestibulopathy patients, and there is a lack of available normative data (4, 15). Few studies have undertaken an objective assessment of gait impairments in both BV and UV patients and no direct comparison between these two patient groups has been performed (16–19). A rigorous and precise method for analyzing individual gait outcomes involves recording spatiotemporal parameters such as walking speed, stride length, and stride time using a motion capture system (20). Previous findings have identified step length variability at slower speeds and step width variability at faster speeds as particularly discriminative parameters between healthy subjects and those with BV (16, 17). While these results are promising, it is important to note that many recent investigations were conducted on treadmills with safety harnesses and imposed walking speeds, rather than overground walking at self-selected paces, thus limiting the representativeness of real-life walking conditions (18). Additionally, some studies were carried out prior to the establishment of the Bárány Society’s diagnostic criteria, potentially affecting the consistency of patient inclusion (17).

Furthermore, morphological variability, particularly differences in participant stature, can have a marked influence on gait parameters (e.g., taller individuals generally have longer steps). To address this, our study uses an adimensionalization approach (normalizing spatiotemporal parameters to leg length), which, to our knowledge, has not been widely implemented in previous work. In addition, many earlier studies pooled subjects ranging from 20 to 80 years old, introducing confounding factors related to normal aging processes (21). An equally important gap is the insufficient exploration of unilateral vestibular (UV) lesions, even though a significant number of UV patients experience persistent gait and balance issues in dynamic conditions long after the acute episode (18, 22). Our investigation aims to fill this void by focusing on both BV and UV populations, applying strict inclusion criteria that align with the Bárány classification. Although this approach yields a relatively small sample size, it enhances the precision of patient characterization and minimizes confounders.

Better understanding of gait disorders in patients with BV and UV is needed to objectively measure the effects of new therapies. Currently, therapeutic approaches mostly consist of physical therapy and supportive strategies like the use of canes and optimizing home ergonomics. While these interventions assume an essential role in patient management, no “curative” treatment is yet available even though encouraging ones are emerging. For instance, galvanic stimulation demonstrated its effectiveness in enhancing the walking ability of patients with BV, particularly at slower speeds, thereby potentially lowering the risk of falls (23). Furthermore, several groups worldwide are currently exploring the therapeutic potential of the vestibular implant (24–27). The underlying concept behind the unilateral vestibular implant is based on the hypothesis that unilateral recovery of vestibular function in BV patients would result in a significant reduction in morbidity. To demonstrate the clinical benefit of the vestibular implant, precise and objective parameters must be strictly defined.

The aim of this study was to investigate gait and symptom severity in individuals with BV and UV. Specifically, we conducted an analysis of spatio-temporal gait parameters and clinical gait tests in BV and UV, and compared them to healthy subjects (HS). To ensure the accuracy and reliability of our results, inclusion criteria of UV and BV patients were in line with diagnostic criteria set by the Bárány Society (4). We hypothesized that spatio-temporal gait parameters would significantly differ in BV patients and that the majority of UV patients would be similar to HS. We also expected higher DHI scores in BV patients compared to UV patients. This would support the idea that unilateral restoration of vestibular function using a vestibular implant in patients with BV could significantly contribute to normalize the gait parameters and, consequently, significantly reduce symptoms.

## Methods

### Participants

Ten BV patients, diagnosed following meticulously the criteria specified by the Classification Committee of the Bárány Society (4), were recruited in a tertiary referral hospital to participate in the study. *A priori* power calculations were not performed; instead, the sample size was determined by the number of patients available during the project’s designated timeframe. Diagnostic criteria for BV included imbalance and/or oscillopsia during walking or head movements, and a reduced bithermal caloric response (sum of bithermal maximal peak slow-phase velocity < 6°/s bilaterally) and/or a bilaterally reduced video head impulse test (vHIT) gain of <0.6, and/or a vestibulo-ocular reflex (VOR) gain <0.1 upon sinusoidal stimulation on a rotatory chair.

Ten UV patients were also recruited using the hospital’s clinical database, with documented unilateral reduced vHIT gain of < 0.6 from at least the lateral canal tested a and normal contralateral vestibular function (vHIT gain values above 0.6), without evidence of other otologic/neurologic disease. UV patients were recruited at least 6 months after acute symptoms. A vHIT was performed before the experiments to ensure that semicircular canal function did not recover in the impaired ear and that the contralateral ear was fully functional.

Finally, ten healthy control subjects (HS) without symptoms of dizziness, vertigo or imbalance were recruited. All HS had normal semicircular canal function with vHIT gain of >0.8 in all canals and no history of otologic/neurologic disease. Subjects were asked by phone to participate in this study and information forms were sent by e-mail or by mail. Subjects with health problems that may affect walking (orthopedic and neurological) were excluded from participation in this study.

The study was designed and conducted in accordance with the guidelines of the Declaration of Helsinki and was approved by the local ethics committee (Commission Cantonale d’Ethique de la Recherche; NAC 11-080 CER 11-219). All subjects provided written informed consent.

### Data collection

#### Acquisition

During gait analysis, participants wore either tight-fitting clothes or only underwear, and were asked to be barefoot. A trained operator collected the participant’s anthropometric data first (i.e., height, body mass, leg length, knee, and ankle widths). The participant was equipped with 35 cutaneous reflective markers (14 mm diameter) attached with double-sided adhesive tape and placed on specific anatomical landmarks according to the Conventional Gait Model 1.0 (28). Three-dimensional kinematics during the different tasks were collected using a 12-camera optoelectronic motion capture system (Oqus 7+, Qualisys AB, Göteborg, Sweden) at an acquisition frequency of 100 Hz. From the markers trajectories, the spatiotemporal parameters were computed.

#### Procedure

The participant was asked to walk back and forth on a 10-meter walkway at three different self-selected speeds: comfortable speed, slow speed, and fast speed. Walking trials at each speed were repeated 3 times.

Measurements were then completed using the 10 tasks of the functional gait assessment battery (FGA) which evaluates walking performance over six meters in different conditions. These tasks were: walking on flat ground (Item 1), changing gait speed (Item 2), walking while performing horizontal and vertical head turns (Items 3 and 4, respectively), walking and making a pivot turn (Item 5), stepping over an obstacle (Item 6), heel-to-toe walking (Item 7), walking with eyes closed (Item 8), walking backwards (Item 9), and walking up and down stairs (Item 10) (29, 30). The participant was first verbally instructed on each task and a demonstration was performed by a trained experimenter. Each item was scored immediately upon completion of each task by the experimenter on a four-point ordinal scale ranging from 0 to 3, with 0 indicating severe impairment (subjective score). The items were then summed to obtain a total score and named subjective score (max. 30), and analyzed by groups individually by task. In addition to these scores, the videos collected by the motion capture system during the FGA were randomized and scored again by two blinded experimenters (randomized score).

The Timed Up and Go (TUG) and Tandem walk were also performed to assess basic functional mobility. The TUG measures the time it takes a participant to stand up from a chair, walk 3 m at a comfortable speed, walk around a cone, walk back, and sit down on the chair (31). Mobility is considered normal if the task can be completed in less than 10 s, between 10 and 20 s the subject is considered to have good mobility, and if the task takes more than 29 s the individual is considered to have impaired mobility. Note that ≥12 s to complete the TUG correlates to a higher risk of falling in community-dwelling older adults (32). For the tandem walk, subjects were asked to walk heel-to-toe for 10 steps at a self-selected pace with their arms crossed over their chest. The number of steps achieved was counted and used as the task score. Finally, the Dizziness Handicap Inventory (DHI) (33), a 25-item, disease specific questionnaire, was completed by UV and BV participants. The higher the score, the greater the perceived handicap due to vestibular symptoms. DHI scores (min 0/max 100) between 16 and 34 indicate a mild handicap, scores between 36 and 52 a moderate handicap, and scores between 54 and 100 a severe handicap. A DHI score of 0 is indicated for HS subjects as they do not exhibit any vestibular symptoms (34).

### Data analysis

#### Data processing

Marker trajectories were labeled with the Qualisys Tracking Manager software (QTM 2019.3, Qualisys, Göteborg, Sweden) and were exported in the c3d file format.^1^ All the processing was performed using Matlab (R2021b, The MathWorks, United States) using the c3d parser Biomechanics Toolkit (BTK) (35). The trajectories were interpolated to fill gaps using reconstruction based on marker inter-correlations (36). Gait events, such as foot strikes and foot offs, were automatically detected using a custom-made algorithm developed in Matlab for self-selection among different methods (37). To prevent detection errors, each event was visually verified by an operator. Then, each 3 × 10-meter trial was divided into gait cycles (foot strike to foot strike) to calculate all the spatio-temporal parameters of this study. We included all valid gait cycles for each participant, resulting in at least two cycles per participant and condition, while also accounting for the potential variability in the number of steps.

#### Spatio-temporal parameters

**Figure 1:**
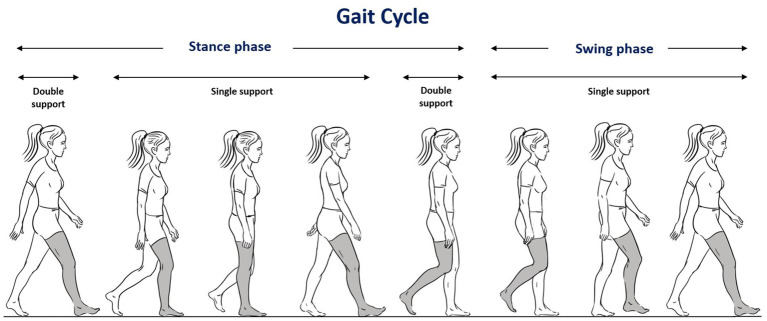
Illustration of the gait cycle (see also Table 1 for detailed descriptions).

Thirteen spatio-temporal parameters were computed and analyzed in this study. The seven most statistically significant parameters are described in Table 1 and Figure 1 (38). Means and standard deviations (SD) of each parameter were determined for each trial, for each participant. The spatio-temporal parameters are inherently associated with the height and body mass of the participants, which could potentially introduce bias in the comparison between groups (39). To compare the HS, UV, and BV groups and to determine if the observed differences were related to pathology, it was necessary to adjust these spatio-temporal parameters for body mass, leg length, and gravity through dimensionless data (40). Note that raw, undimensionalized data are also provided as Supplementary material.

**Table 1:** Gait parameters of interest and their definition.

Gait parameters	Definition
Spatial parameters	
Step length (m)	Anterior–posterior distance from the heel of one footprint to the heel of the opposite footprint.
Step width (m)	Lateral distance from heel of one footprint to the line of progression formed by two consecutive footprints of the opposite foot.
Temporal parameters	
Cadence (steps/min)	Number of steps per minute.
Spatiotemporal parameters	
Walking speed (m/s)	Distance walked divided by the ambulation time.
Temporophasic parameters	
Stance phase (%GC)	The weight bearing portion of each gait cycle initiated at heel contact and ending at toe off of the same foot.
Single support (%GC)	Only one foot is in contact with the ground during the time elapsed between the last contact of the opposite footfall and the initial contact of the next footfall of the same foot, normalized to stride time.
Double support (%GC)	Both feet are in contact with the ground simultaneously during the sum of the time elapsed during two periods of double support in the gait cycle, normalized to stride time.

m, meters; min, minute; s, seconds; %GC, % gait cycle.

### Statistical analysis

IBM SPSS Statistics version 26 (Armonk, New York, United States) and SigmaPlot 14.5 (Systat Software, San Jose, CA, United States) were used for data analysis. After verifying normal distribution of the data (Shapiro–Wilk test) Kruskal–Wallis one way analysis of variance and post-hoc pairwise comparisons (Dunn’s test) were employed, to statistically compare parameters the clinical gait tests (FGA, TUG, Tandem walk and DHI) between all 3 groups (HS/BV/UV). A mixed-model repeated measures analysis of variance was then conducted to assess differences in spatio-temporal gait parameters between groups and at each walking speed. In this analysis, each valid gait cycle constituted a unit of observation, and a random effect was assigned to each participant, thereby preserving within-subject variability. To control the false discovery rate, *p*-values were adjusted with a Classical one-stage method (41). Finally, a two-step cluster analysis was used to identify subgroups within the patients. This statistical test is a hybrid method that initially employs a distance measure to differentiate groups, followed by a probabilistic approach to determine the best-fitting subgroup model (42). The significance threshold for the statistical tests was set at 0.05.

## Results

### Demographic data

A total of 30 participants were included in the study: 10 HS [6 women, mean age: 64.6 years; (SD): 10.02], 10 UV [5 women, mean age: 63.4 years; (SD): 6.22] and 10 BV[5 women, mean age: 64.4 years; standard deviation (SD): 9.61]. Demographics and anthropomorphic data are summarized in Table 2 and etiologies in Table 3. Mean Height and body mass index, 1.71 m [0.71] and 24.24 [3.55] for HS, 1.68 m [0.16] and 27.91 [2.38] for UV and were 1.65 m [0.90] and 26.41 [3.78] for BV, respectively. There were no significant differences in the demographic and anthropomorphic data between the three groups (*p* > 0.05).

**Table 2:** Main demographic and anthropomorphic characteristics of the 30 patients included in this study.

	HS	UV	BV	Statistic (overall group factor) *p* values
Participant number (*n*)	10	10	10	
Sex (*n*)				
Female	6	5	5	–
Male	4	5	5	–
Age, years old (mean, SD)	64.6 (10.02)	63.4 (6.22)	64.4 (9.61)	0.977
Height (m)	1.71 (0.71)	1.68 (0.16)	1.65 (0.90)	0.432
BMI (kg/m^2^)	24.24 (3.55)	27.91 (2.38)	26.41 (3.78)	0.060

*n, number of subjects; SD, standard deviation; m, meter; BMI, body mass index; p-values indicate results of the ANOVA analysis.*

**Table 3:** Etiological data for bilateral (BV) and unilateral (UV) vestibulopathy patients in this study.

Patients	Sex	Affected side	Etiology	Score DHI
BV				
1	M	Both	Schwannoma	2
2	F	Both	Ototoxic	48
3	M	Both	Idiopathic	48
4	F	Both	Idiopathic	20
5	F	Both	Idiopathic	34
6	F	Both	Idiopathic	74
7	M	Both	Idiopathic	40
8	F	Both	Idiopathic	NA
9	F	Both	Genetic (DFNA9)	46
10	M	Both	Idiopathic	12
UV				
1	F	L	Idiopathic	68
2	M	R	Idiopathic	8
3	M	L	Schwannoma	20
4	M	R	Traumatic	11
5	M	L	Idiopathic	6
6	M	L	Post-labyrinthectomy	64
7	F	R	Schwannoma	52
8	F	R	Idiopathic	2
9	F	R	Idiopathic	14
10	F	R	Idiopathic	22

Sex = F, Female; M, Male; Affected side = L, Left; R, Right; DHI, dizziness handicap inventory; NA, Not available.

### Clinical gait assessments

The outcomes of the FGA, TUG, tandem walk and DHI are shown in Table 4. Kruskal–Wallis revealed significant differences in FGA scores between the three groups. Median (min-max) randomized scores were 29 (22–30), 27.3 (11.5–29.5) and 19.5 (13.5–24) for HS, UV and BV, respectively (note that lower scores indicate poorer performance). The different items of the FGA were analyzed individually (Supplementary Table S1). In BV patients, a score of 2 was observed for walking on a level surface and walking with horizontal head turns, while scores of 0 and 0.5 were recorded for tandem walking and walking with eyes closed, respectively. For all other tasks, BV patients achieved a scores of 3. In UV patients, a score of 2 was recorded for walking on a level surface, and a score of 1.5 for walking with eyes closed. Among HS, scores ranged between 2.5 and 3 across all tasks.

**Table 4:** Clinical gait assessments.

	HS	UV	BV	Statistic (*p* values)	*Post-hoc* sig.		
	Median (min-max)	Median (min-max)	Median (min-max)		HS vs. BV	HS vs. UV	UV vs. BV
Functional scores							
FGA (subjective score)	29 (27–30)	27.5 (8–30)	19 (15–29)	**<0.001**	**<0.001**	0.102	0.141
FGA (randomized score)	29 (22–30)	27.3 (11.5–29.5)	19.5 (13.5–24)	**0.001**	**<0.001**	0.345	0.114
Timed Up and Go (s)	9 (7.5–10.5)	10 (8.5–15.5)	10 (8.5–14.5)	0.1			
Tandem walk (step number)	10 (4–11)	10 (2.5–10.5)	0.75 (0–3)	**<0.001**	**<0.001**	0.98	**<0.001**
Vestibular symptoms score							
DHI (score)		27 (2–68)	36 (2–74)				

s, second; m/s, meter per second; min, minimum; max, maximum; *p*-values indicate results of Kruskal–Wallis One Way Analysis of Variance and post-hoc tests (Dunn’s test). Significant differences are highlighted in bold.

The difference was statistically significant (Dunn’s test) between HS and BV (*p* < 0.001). No statistically significant differences were found for TUG scores (*p* = 0.1). We also observed significant differences in the number of steps for the tandem walk task (*p* < 0.001). Post-hoc comparisons revealed significant differences (*p* < 0.001) between the UV [10 (2.5–10.5) steps] and BV [0.8 (0–3) steps] and also between HS [10 (4–11) steps] and BV. DHI scores were 27 (2–68) in UV and 36 (2–74) in BV subjects.

### Dimensionless spatio-temporal gait parameters

**Figure 2:**
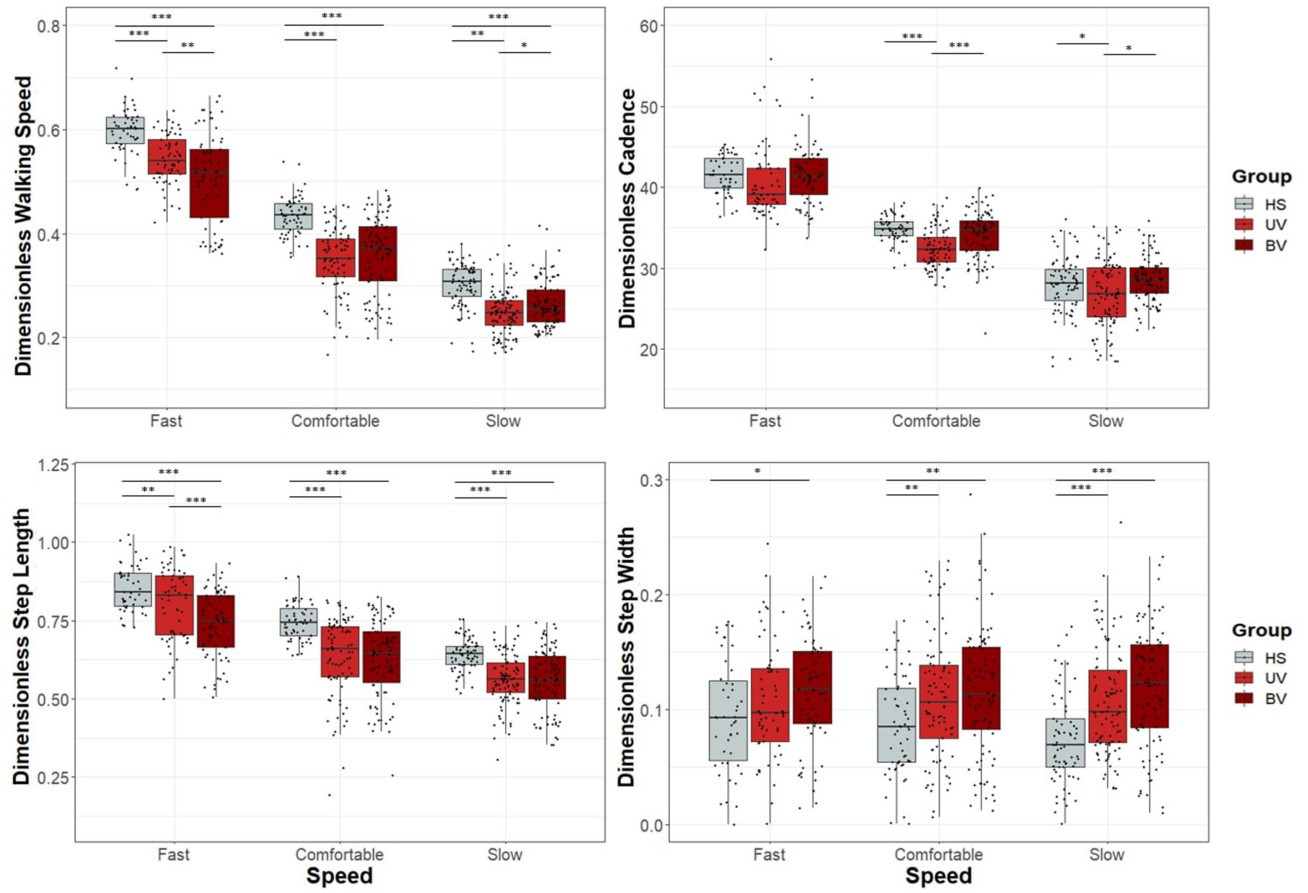
Box and dot plots of walking speed, cadence, step length and step width across three self-selected walking speeds (fast on the left, comfortable on the middle and slow on the right) in healthy (HS) (gray), unilateral vestibulopathy (UV) (light red), and bilateral vestibulopathy (BV) (dark red) groups. Dimensionless values are presented. Box plots indicate median values, 25th and 75th percentile values (colored box) as well as minimum and maximum values (error bars). A point corresponds to the individual results of one trial of subject. Stars and black horizontal lines indicate significant differences between groups for each speed (^*^p < 0.05, ^**^p < 0.01, ^***^p < 0.001).

Descriptive dimensionless data for seven spatio-temporal gait parameters, are presented for the three groups (HS, UV, and BV) in Table 5 and illustrated in Figure 2. Mean (HS, UV, and BV) slow speeds were 0.30, 0.25 and 0.26; comfortable speeds were 0.43 0.35 and 0.36; and fast speeds were 0.60, 0.54 and 0.51. All three self-selected speeds were significantly different between the three groups (*p* < 0.001). UV and BV subjects had slower dimensionless walking speeds than HS in all conditions (*p* < 0.001, Table 5 and Figure 2). Significant differences were also found between UV and BV subjects at slow (*p* = 0.019) and fast speeds (*p* = 0.003). All dimensionalized spatiotemporal gait parameters are available in Supplementary Table S2.

**Table 5:** Dimensionless spatio-temporal gait parameters (mean value ± SD) for HS, UV and BV participants.

Self selected speed	ST parameter	Groups						Mixed-model	*Post-hoc* sig.		
		HS		UV		BV					
		Mean	±SD	Mean	±SD	Mean	±SD	*p* value	HS vs. BV	HS vs. UV	UV vs. BV
Slow											
	Walking speed	0.30	0.04	0.25	0.04	0.26	0.04	**0.000**	0.000	0.000	0.019
	Cadence	28.03	3.41	26.83	4.05	28.57	2.78	**0.002**	0.312	0.023	0.001
	Step length	0.64	0.05	0.56	0.08	0.56	0.10	**0.000**	0.000	0.000	0.800
	Step width	0.07	0.04	0.11	0.05	0.12	0.05	**0.000**	0.000	0.000	0.062
	Stance phase (%GC)	63.50	2.60	66.89	2.78	65.10	2.87	**0.000**	0.000	0.000	0.000
	Double support (%GC)	27.58	4.15	33.25	4.57	30.53	4.64	**0.000**	0.000	0.000	0.000
	Single support (%GC)	35.65	2.18	33.65	2.83	34.57	3.05	**0.000**	0.013	0.000	0.020
Comfortable											
	Walking speed	0.43	0.04	0.35	0.06	0.36	0.08	**0.000**	0.000	0.000	0.467
	Cadence	34.89	1.62	32.41	2.37	34.12	2.94	**0.000**	0.062	0.000	0.000
	Step length	0.75	0.06	0.64	0.13	0.63	0.12	**0.000**	0.000	0.000	0.575
	Step width	0.09	0.04	0.11	0.06	0.11	0.06	**0.005**	0.002	0.006	0.753
	Stance phase (%GC)	61.43	1.79	63.65	2.66	62.91	2.35	**0.000**	0.000	0.000	0.038
	Double support (%GC)	23.66	3.25	27.50	4.58	25.46	4.47	**0.000**	0.014	0.000	0.002
Fast											
	Walking speed	0.60	0.05	0.54	0.05	0.51	0.09	**0.000**	0.000	0.000	0.003
	Cadence	41.53	2.31	40.73	4.64	41.48	3.55	0.411			
	Step length	0.86	0.08	0.80	0.12	0.74	0.10	**0.000**	0.000	0.010	0.000
	Step width	0.10	0.05	0.11	0.05	0.12	0.05	0.068			
	Stance phase (%GC)	59.99	1.50	60.31	2.06	60.74	2.83	0.193			
	Double support (%GC)	20.34	2.63	21.66	3.48	21.49	5.23	0.208			
	Single support (%GC)	39.53	1.68	38.64	1.97	39.25	3.10	0.143			

m, meters; min, minute; s, seconds; ST, spatiotemporal; SD, standard deviation; %GC, % Gait Cycle, 0.000, 0.0001. Significant differences are highlighted in bold. For the mixed-model analysis and in red for post-hoc tests.

For all three walking speeds, BV subjects had significantly shorter step length (*p* < 0.001) and significantly broader step width (*p* < 0.001) but similar cadence to HS (*p* > 0.01). Similarly, UV subjects had shorter step length (*p* < 0.001) at all speeds compared to HS. For the fast walking speed, the step length for UV subjects was significantly smaller than that of HS (*p* < 0.001), although their cadence did not significantly differ (*p* = 0.411). Compared to HS, UV participants had significantly narrower step width (*p* < 0.001) with slower cadence (*p* < 0.01) at slow and comfortable walking speeds.

**Figure 3:**
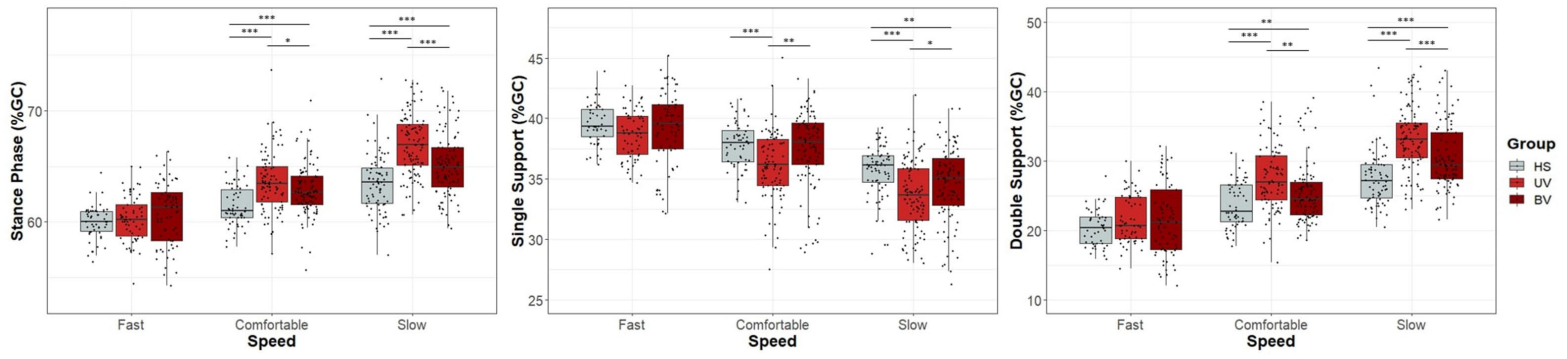
Box and dot plots of stance phase, single support and double support across three self-selected walking speeds (fast, comfortable, and slow) in healthy (HS) (gray), unilateral vestibulopathy (UV) (light red), and bilateral vestibulopathy (BV) (dark red) groups. Box plots indicate median values, 25th and 75th percentile values (colored box) as well as minimum and maximum values (error bars). A point corresponds to the individual results of one trial of one subject. Stars and black horizontal lines indicate significant differences between groups for the indicated speed (^*^p < 0.05, ^**^p < 0.01, ^***^p < 0.001).

Stance phase was longer in UV and BV subjects at comfortable (63.65 ± 2.66%, *p* < 0.001 for UV and 62.91 ± 2.35%, *p* < 0.001 for BV) and slow walking speeds (66.89 ± 2.78% *p* < 0.001 for UV and 65.10 ± 2.87%, *p* < 0.001 for BV) compared to HS participants (61.43 ± 1.79 and 63.50 ± 2.60% for comfortable and slow walking speeds respectively; see Table 5 and Figure 3). No significant difference was found between groups at fast walking speed (*p* = 0.193). Results also showed differences between UV and BV subjects at comfortable (*p* = 0.038) and slow walking speeds (*p* < 0.001).

The single support phase was shorter in UV and BV subjects at slow walking speed (33.65 ± 2.82, *p* < 0.001 for UV and 34.57 ± 3.05, *p* = 0.013 for BV) compared to HS participants (35.65 ± 2.18). At comfortable walking speed, significant differences were found only between UV (36.14 ± 2.85) and the other groups (37.45 ± 3.24, *p* = 0.002 for BV and 37.79 ± 1.94, *p* < 0.001 for HS).

Double support phase was longer between both pathological groups (UV and BV) compared to HS at comfortable (UV: 27.50 ± 4.59, *p* < 0.001 and BV: 25.46 ± 4.47, *p* = 0.014) and slow (UV: 33.25 ± 4.57, *p* < 0.001 and BV: 30.53 ± 4.64, *p* < 0.001) walking speeds. At fast walking speed, there was no significant difference between groups for single and double support phase.

**Figure 4:**
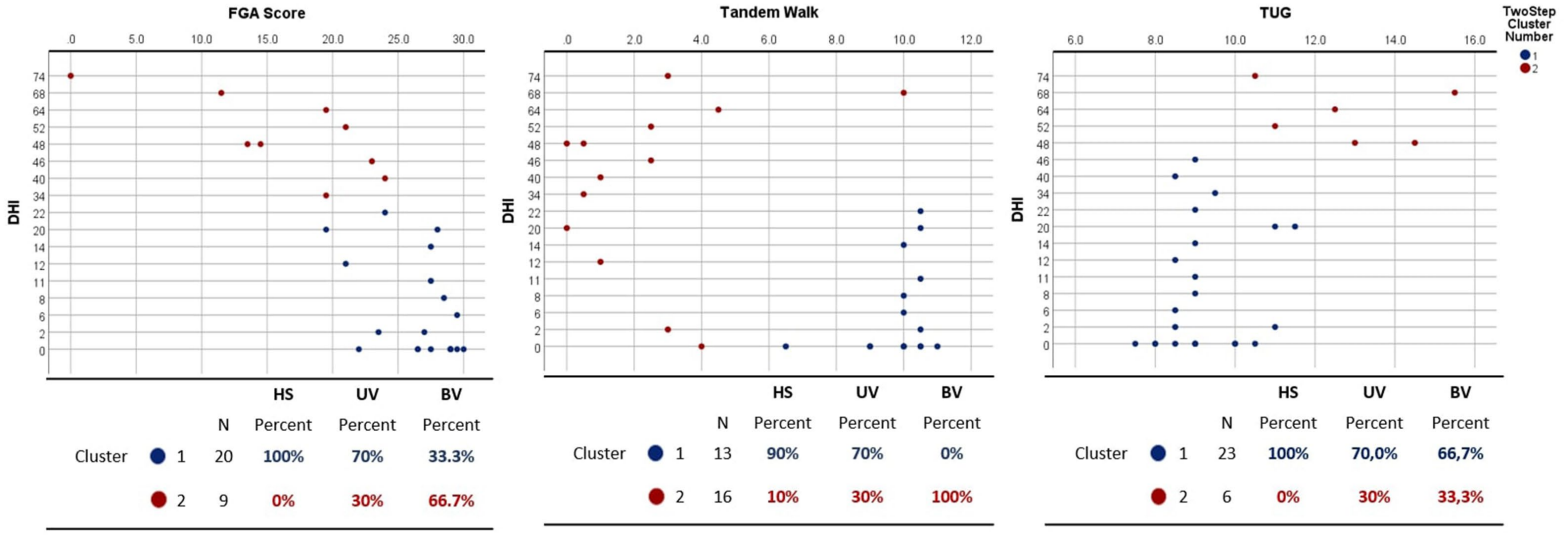
Two-step cluster analysis comparing dizziness handicap inventory (DHI) with functional gait assessment (FGA) score (left panel), Tandem walk (middle panel) and Timed Up and Go (TUG) (right panel) for healthy (HS), unilateral (UV) and bilateral (BV) vestibulopathy subjects. Two clusters (in red and blue) were obtained. Tables below illustrate the distribution of patients across clusters, delineating the corresponding proportions of individuals in the HS, UV, and BV cohorts.

A two-step cluster analysis was used to compare patient-reported symptoms through validated questionnaire (DHI) with clinical functional tests (Figure 4) and spatio-temporal gait parameters (Supplementary Table S3). Two clusters were obtained by comparing DHI and FGA score, tandem walk, and TUG for each study participant.

Cluster 1 (in blue) had better (lower) scores for DHI and better (higher) FGA scores (between 0 and 22 for DHI and between 20 and 30 for FGA) with 100% of HS, 70% of UV, and 33.3% of BV. Cluster 2 (in red) score demonstrated very poor DHI scores (between 34 and 74) with poor FGA scores (between 0 and 23). This cluster contained 30% of UV and 66.7% of BV.

For DHI and tandem walk the first cluster exhibits better DHI scores, ranging from 0 to 22, and a higher number of steps, between 7 and 11. The second cluster presents a wide range of DHI scores, varying from 0 to 74, with a relatively small number of steps in the tandem walk, except for one subject, with high DHI score (74) and 10 steps. This cluster contained 100% of the BV, 30% of the UV, and 10% of the HS.

Regarding the TUG, cluster 1 comprises the majority of participants, with 100% HS, 70% UV, and 66.7 BV, presenting a wide variety of DHI scores ranging from 0 to 46 and TUG results of 7 to 11 s. The second cluster has only 6 values with very poor DHI scores ranging from 48 to 74 and a longer TUG between 10 and 15 s. This cluster contained 33% of the BV, 30% of the UV.

## Discussion

The aim of this study was to investigate the impact of chronic unilateral and bilateral loss of vestibular function on clinical gait assessment and spatio-temporal parameters of gait during ground walking at three self-selected speeds (slow, comfortable, and fast). We observed significant differences in FGA between groups, with pathological scores only on specific tasks. We also observed moderate to severe self-reported handicap according to DHI scores for UV and BV. However, no significant differences were observed for the TUG test. Regarding spatio-temporal parameters, BV and UV walked slower but with similar cadence to HS at all three self-selected speeds. They had significantly shorter step length and broader step width, which varied with walking speed. Individuals with vestibular pathologies also exhibited a longer stance and double support phase and a shorter single support phase than HS, which also varied with walking speed. The hypothesis that the spatio-temporal gait parameters would significantly differ in BV patients and that the majority of UV patients would be similar to HS was therefore confirmed. The cluster analysis between clinical functional tests (FGA, tandem walk, and TUG), spatio-temporal gait parameters and their symptoms through DHI revealed two clusters, with one containing all HS and the majority of UV (70%), and another cluster with a majority of BV and a small proportion of UV (30%).

### Clinical gait assessments

The negative impact of chronic bilateral vestibular vestibulopathyon daily activities is evident in quality-of-life scores, particularly in terms of physical functioning (14). It is plausible that the observed restrictions are significantly influenced by postural instability and gait disorders, which in turn limit physical activity and promote an increase in sedentary behavior, associated with various detrimental health effects, such as hypertension and obesity (43). Consequently, the reduction in overall physical activity could be an independent risk factor for the development of serious comorbidities in patients with BV.

Clinical tests like the FGA and TUG tests are crucial for identifying fall-related disorders and prescribing appropriate interventions (44, 45). In this study, FGA average scores allowed us to differentiate BV and UV, but there was significant variability within these groups, with very different min-max values. The analysis of individual FGA items highlighted task-specific impairments in both BV and UV patients, which are representative of their reported difficulties in daily life. Interestingly, BV patients exhibited mild impairments in walking on a level surface and with horizontal head turns, while severe deficits were observed in more demanding tasks, such as tandem walking and walking with eyes closed. These findings align with patient complaints of significant instability in conditions requiring precise postural control or reduced sensory input.

Similarly, in UV patients, only walking on a level surface and walking with eyes closed showed mild to moderate deficits, suggesting that while their overall gait remains functional, they experience noticeable difficulties in specific conditions. This pattern reflects the subjective experiences of UV patients, who often report challenges in maintaining stability under conditions of altered visual or proprioceptive feedback. The TUG test is a tool used in vestibular physiotherapy to assess walking and turning ability. It has an optimal score of 13.5 s and can predict falls in the next 6 months in elderly adults (31, 46, 47). Even though no significant difference was observed between HS and the pathological groups, the vast majority of subjects (87%) had a score below 13.5 s with only 3 out of 30 subjects exceeding the optimal threshold and presenting a risk of falling. Therefore, even if it might give a good idea of the most severe cases, the TUG test may not be adapted to this population as it solely gives an indication on the required time to complete the task but does not thoroughly assess turning abilities. Finally, the tandem walk appears to be a sensitive and highly discriminating test for detecting patients with severe vestibular deficits. BV patients are able to perform 1.2 steps compared with 8.9 steps for UV and 9.05 steps for HS. Tandem walk thus appears as a useful and valuable diagnostic tool, especially for clinicians, as it is easy to administer and quickly provides a good overview of the subject’s dynamic balance. In case of doubt during the test, performing it with eyes closed can be even more discriminating (48).

### Spatio-temporal gait parameters

This study builds upon previous observations regarding gait impairments in BV patients, highlighting that certain measured spatio-temporal parameters significantly differ, which may indicate an increased risk of falling (16, 17, 49). Consistent with these observations, our study confirmed that individuals suffering from chronic vestibulopathy, either bilateral (BV) or unilateral (UV), exhibited a lower preferential gait speed, along with increased step width and a prolonged double stance phase. These findings align with common clinical observations of “cautious” gait characterized by wider, shorter steps. Alterations in the base of support might also help patients better manage their center of mass, and thus their postural stability. This supports the notion that vestibular inputs are necessary to maintain the gait pattern and refine foot movements to sustain dynamic stability and maintain intended paths (16, 50).

Our study also found that, at fast walking speeds, significant differences between the groups in terms of step width, stance, single, and double support phases disappeared. Hence, this condition may not be suitable for objective outcomes but rather serves as a valuable experimental setting. In other words, exploring the relationship between walking speed, imbalance, and vestibular symptoms in individuals with vestibulopathy is a subject of great interest as patients exhibit more instability during slow walking speed (51). An interesting study showed that a dog with acute unilateral vestibulopathy exhibited less imbalance when running than when walking (52). This suggests that vestibular signals appear to contribute to the maintenance of balance during locomotion, but this influence decreases as walking speed increases (53). Patients with BV have been found to exhibit higher variability in most parameters during slow walking. Two possible explanations for this phenomenon exist: firstly, it is possible that increased walking speed results in passive mechanical stabilization of balance (54). Then, it is possible that vestibular input into the sensorimotor control of locomotion is partially suppressed with increasing walking pace and speed, leading to less contribution of vestibular inputs on lower limb muscles (53, 55, 56). Some studies have found that increased gait fluctuations during slow walking are the most predictive factor of an increased risk of falling (49). Taken together, the results provide objective and specific outcomes that can guide clinicians in assessing and monitoring the severity of vestibulopathy and, importantly, to objectively measure the effects of new therapies.

### DHI and patients’ symptoms

The DHI scores for UV subjects were found to be 36 (2–74), while BV subjects had scores of 27 (2–68). This result shows that, as a group, UV subjects experienced mild disability, while BV subjects had moderate disability (57). However, it is important to note that both groups had a wide range of DHI scores, indicating a high degree of symptom variability among patients. Previous studies have unsuccessfully attempted to establish a correlation between DHI scores and clinical vestibular tests (58). Several explanations have been proposed, such as the DHI’s lack of sensitivity, the lack of correlation between a patient’s perception of disability and their real deficits, and the influence of factors like central compensation, adaptation, and sensory rebalancing. Given the high variability among subjects and the small number of subjects, it may not be relevant to search for a correlation. Instead, it might be more valuable to examine the distribution of patients and identify any patterns or potential clusters, both within and between groups. Cluster analysis and subject distribution revealed two subgroups, one containing all HS and the majority of UV, and the other including a large majority of BV and a small proportion of UV. Upon closer examination, the findings of the BV group appear quite consistent, displaying a range of symptoms that align with clinical observations. The DHI, a subjective measurement, appears to be influenced by the chronic nature of the disease, the various compensatory strategies implemented over the years, and the wide diversity of etiologies present in this patient group. In the UV group, we noticed that the majority of individuals performed similarly to healthy subjects. However, three UV patients seemed particularly affected by unilateral vestibulopathy, evident through their symptoms and poor performance in all assessments (6). The persistence of symptoms in these three patients could be multifactorial, including factors such as etiology (e.g., post-labyrinthectomy, vestibular schwannoma) or inadequate central compensation (contralateral spontaneous beating nystagmus, grade III nystagmus). However, given the limited number of patients, drawing definitive conclusions is challenging. Therefore, we conclude that despite these 3 particular UV cases, our results are consistent with our initial hypothesis that restoring vestibular function on one side in patients with BV (turning them into UV patients) could improve objective and subjective symptoms, bringing them closer to the healthy group.

### Study limitations and perspectives

This study has several limitations. The primary limitation of this study is the small number of participants in each group (10) and the limited number of trials per condition. The low participant count is due to the prevalence of this pathology and our adherence to the Bárány Society’s (4) selection criteria to ensure accurate diagnosis and precise characterization of severe vestibulopathy (59, 60). Using less stringent criteria could have increased the cohort size. It is also important to note that other studies in this field have used even smaller sample sizes. Consequently, we believe that this dataset is valid and offers a valuable baseline for future research involving larger cohorts. We aimed to ensure the gait was as natural as possible by allowing participants to choose their walking speeds based on their understanding of the instructions. As a result, spontaneous speeds varied among subjects, groups, and trials, unlike the uniform speeds often observed when using treadmills. It is noteworthy that other studies (16, 18, 61) on similar populations have not reported such large differences in self-selected walking speeds between groups [e.g., 1.00 ± 0.18 m/s for patients with bilateral vestibulopathy versus 1.11 ± 0.19 m/s for healthy subjects (61)]. This discrepancy is likely due to the conditions under which the data were acquired, such as walking on a treadmill.

Secondly, vestibular impairments result in gait disorders, but there are several other age-related deficits that can also contribute. These include other sensory deficits such as vision, somatosensory, and hearing, as well as neuromotor and musculoskeletal deficits, cognitive deficits, and emotional and psychological factors (62–64). These risk factors can worsen the impact of vestibular impairment on balance control and/or interact as confounding factors. To improve clinical care and objectively quantify the effects of new therapeutic interventions such as vestibular implants, it is essential to determine if a person’s balance problem is at least partially caused by their vestibular impairment rather than other coexisting disabilities by evaluating all the functions (e.g., neuropsychological tests, and psychological assessments). Additionally, in our study we have observed significant improvements in some patients’ performance through lifestyle changes like exercise or vestibular physiotherapy practice, for which we did not control but need to be considered in future studies. In this study, we prioritized the quality of diagnosis over the quantity of participants.

The clinical implementation of these techniques is highly dependent on accurate measurements. Therefore, developing protocols using portable inertial sensors capable of accurately delivering significant gait metrics during daily activities would be essential for future projects. The tasks performed during this study were relatively simple (walking 10 meters on a uniform surface with good lighting). Future studies should likely assess walking function in experimental settings more closely aligned with patient complaints, such as walking on uneven surfaces, with reduced lighting, or dual-task conditions, to uncover subtle deficits in patients with vestibular disorders.

## Conclusion

In conclusion, we showed notable differences between groups in terms of FGA scores, tandem walk, and a subset of spatiotemporal parameters at different walking speeds. Participants with vestibular impairments had slower walking speeds, shorter step lengths, and broader step widths in comparison to healthy individuals. Furthermore, they also had different stance, double and simple support phases during slow and comfortable walking speeds. This research emphasizes the effects of an altered vestibular system on clinical gait assessments and walking patterns, providing valuable outcomes for the clinical assessment patients affected with vestibular disorders. The objective measures highlighted in this study, such as gait parameters and FGA scores, could serve as valuable tools for evaluating the effectiveness of vestibular implants and other rehabilitation strategies.

## Data Availability

The datasets presented in this study can be found in online repositories. The names of the repository/repositories and accession number(s) can be found at: https://doi.org/10.5281/zenodo.14236617.

## Footnotes

^1^ https://www.c3d.org
