# Supplementary figures and images for: Gait impairments in patients with bilateral vestibulopathy and chronic unilateral vestibulopathy
Source: Front Neurol. 2025 Feb 27;16:1547444. doi: 10.3389/fneur.2025.1547444 (PMC11903280; doi:10.3389/fneur.2025.1547444)

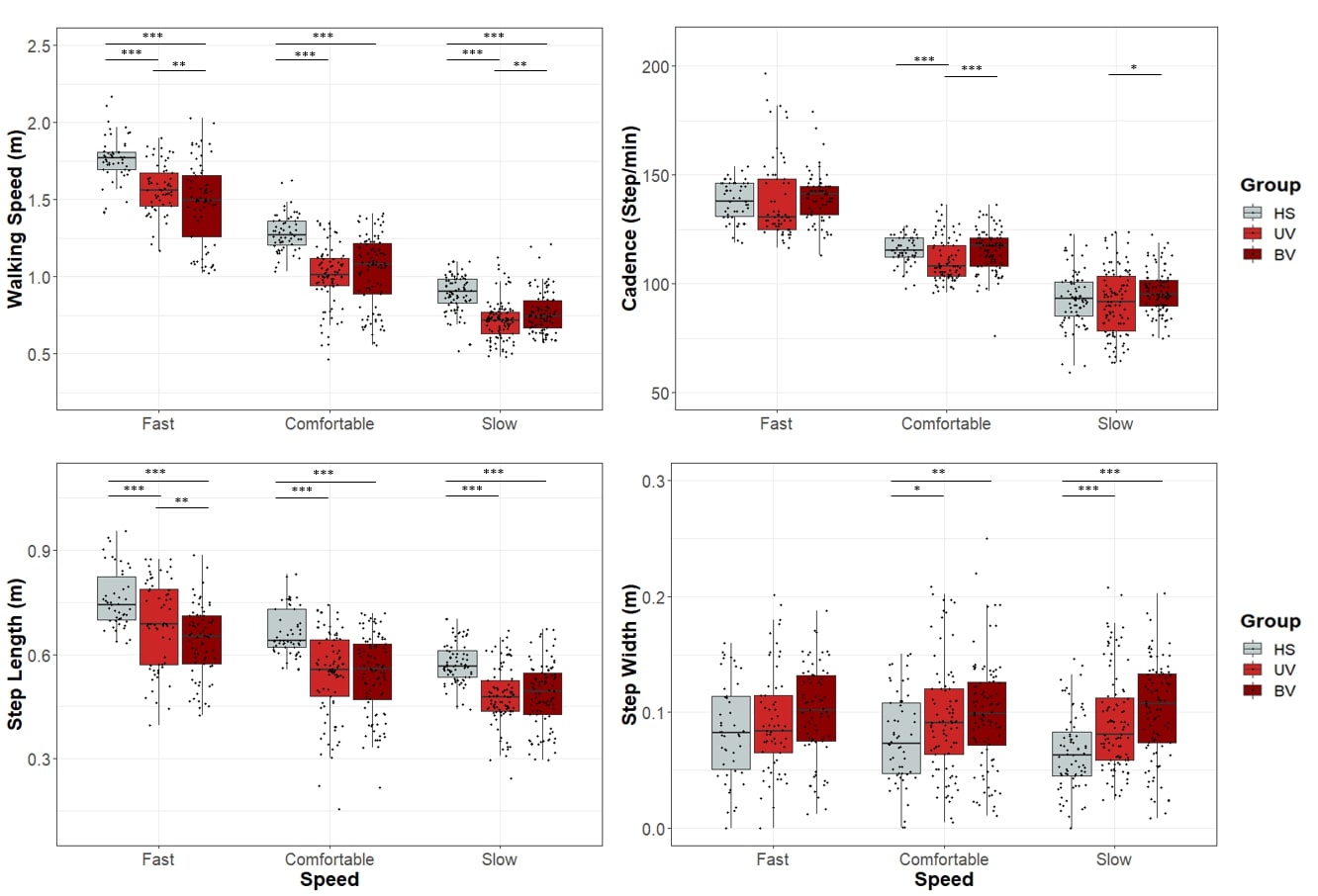

Supplement: Supplementary file 1 [file Supplementary_file_1.jpeg]
