# Supplementary material for: Gait impairments in patients with bilateral vestibulopathy and chronic unilateral vestibulopathy
Source: Front Neurol. 2025 Feb 27;16:1547444. doi: 10.3389/fneur.2025.1547444 (PMC11903280; doi:10.3389/fneur.2025.1547444)
Supplement: Supplementary file 2 [file Table_1.docx]

Supplementary Material

# Supplementary Data


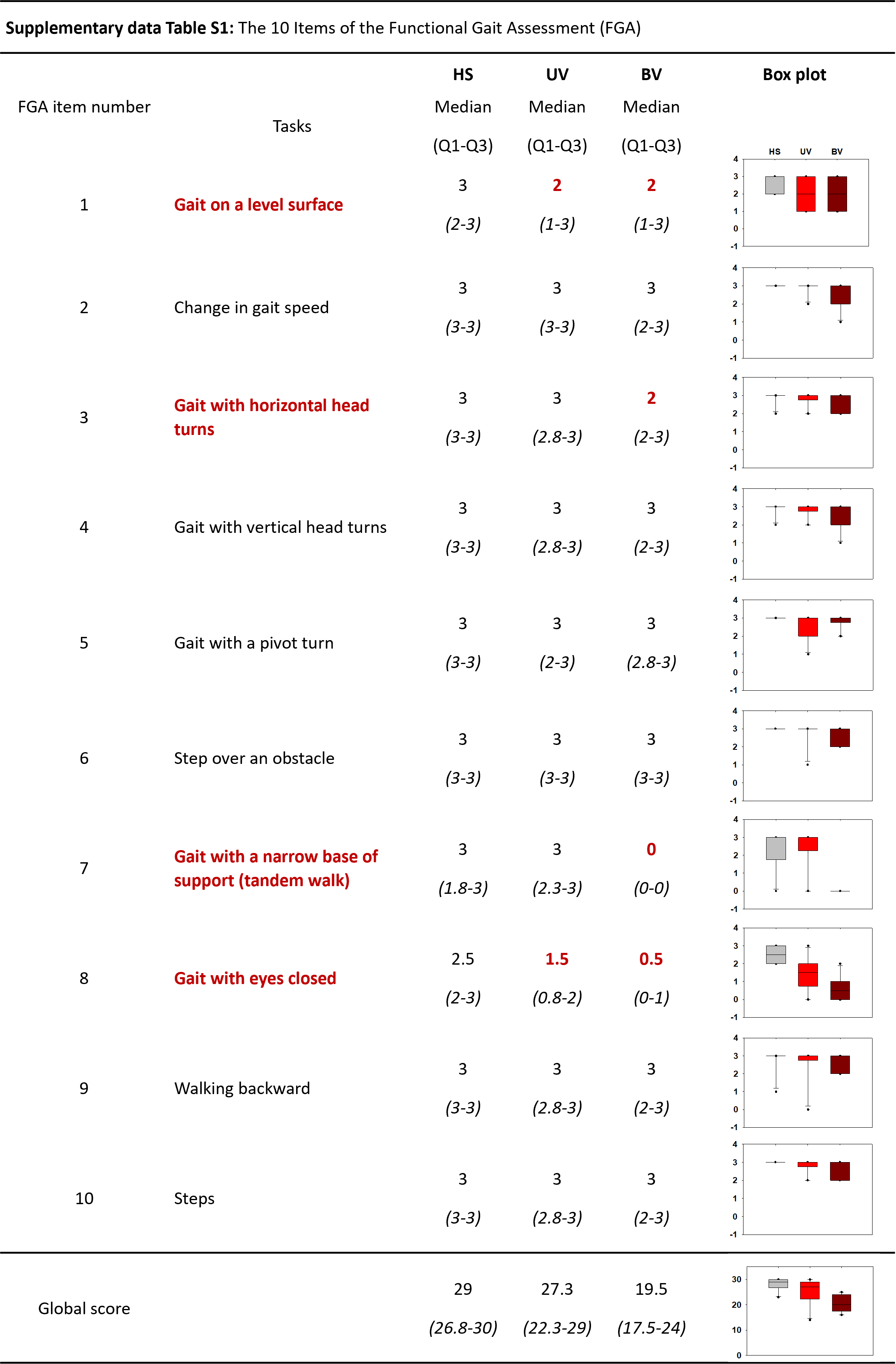


***Note:*** *A score of 3 indicates normal performance, 2 corresponds to mild impairment, 1 to moderate impairment, and 0 to severe impairment or inability to perform. Scores and tasks ranging from mild to severe impairment are highlighted in red.* *The 25th percentile (Q1) and the 75th percentile (Q3) are represented and the box plot is illustrated on the left.*

| **Supplementary data Table S2:** Spatiotemporal gait parameters (mean value ± SD) in HS, UV and BV sujects (data dimensionalized). | | | | | | | | | | | |
| --- | --- | --- | --- | --- | --- | --- | --- | --- | --- | --- | --- |
|  |  | **Groups** | | | | | | Mixed  model | Post Hoc Sig. | | |
|  |  | **HS** | | **UV** | | **BV** | |  |  |  |  |
|  |  | Mean | ±SD | Mean | ±SD | Mean | ±SD | p value | HS vs BV | HS vs UV | UV vs BV |
| **Self selected speed** | **ST Parameter** |  | | | | | |  |  |  |  |
| Slow | | | | | | | | | | | |
|  | Walking_speed (m/s) | 0.89 | 0.12 | 0.72 | 0.13 | 0.77 | 0.14 | 0.000 | 0.000 | 0.000 | 0.007 |
|  | Cadence (step/min) | 92.10 | 12.30 | 91.54 | 15.13 | 96.29 | 9.89 | 0.032 | 0.092 | 0.449 | 0.010 |
|  | Step Length (m) | 0.57 | 0.06 | 0.48 | 0.08 | 0.48 | 0.09 | 0.000 | 0.000 | 0.000 | 0.675 |
|  | Step_Width (m) | 0.07 | 0.03 | 0.09 | 0.04 | 0.10 | 0.04 | 0.000 | 0.000 | 0.000 | 0.071 |
|  | Stance phase (%GC) | 63.49 | 2.60 | 66.89 | 2.78 | 65.10 | 2.87 | 0.000 | 0.000 | 0.000 | 0.000 |
|  | Double Support (%GC) | 27.58 | 4.15 | 33.25 | 4.57 | 30.53 | 4.64 | 0.000 | 0.000 | 0.000 | 0.000 |
|  | Single Support (%GC) | 35.65 | 2.18 | 33.65 | 2.83 | 34.57 | 3.05 | 0.000 | 0.013 | 0.000 | 0.020 |
| Comfortable | | | | | | | | | | | |
|  | Walking_speed (m/s) | 1.29 | 0.12 | 1.01 | 0.19 | 1.04 | 0.23 | 0.000 | 0.000 | 0.000 | 0.302 |
|  | Cadence (step/min) | 115.81 | 6.24 | 110.61 | 9.68 | 115.07 | 9.65 | 0.001 | 0.625 | 0.001 | 0.001 |
|  | Step_Length (m) | 0.66 | 0.07 | 0.54 | 0.12 | 0.54 | 0.11 | 0.000 | 0.000 | 0.000 | 0.960 |
|  | Step_Width (m) | 0.08 | 0.04 | 0.10 | 0.05 | 0.10 | 0.05 | 0.022 | 0.009 | 0.021 | 0.746 |
|  | Stance phase (%GC) | 61.43 | 1.79 | 63.65 | 2.66 | 62.91 | 2.35 | 0.000 | 0.000 | 0.000 | 0.038 |
|  | Double Support (%GC) | 23.66 | 3.25 | 27.50 | 4.58 | 25.46 | 4.47 | 0.000 | 0.014 | 0.000 | 0.002 |
|  | Single Support (%GC) | 37.79 | 1.94 | 36.14 | 2.85 | 37.45 | 3.24 | 0.001 | 0.488 | 0.001 | 0.002 |
| Fast | | | | | | | | | | | |
|  | Walking_speed (m/s) | 1.76 | 0.15 | 1.57 | 0.16 | 1.48 | 0.27 | 0.000 | 0.000 | 0.000 | 0.013 |
|  | Cadence (step/min) | 137.88 | 8.81 | 138.68 | 18.80 | 139.96 | 11.16 | 0.701 |  |  |  |
|  | Step_Length (m) | 0.76 | 0.09 | 0.69 | 0.13 | 0.64 | 0.10 | 0.000 | 0.000 | 0.001 | 0.009 |
|  | Step_Width (m) | 0.08 | 0.04 | 0.09 | 0.04 | 0.10 | 0.04 | 0.144 |  |  |  |
|  | Stance phase (%GC) | 59.99 | 1.50 | 60.31 | 2.06 | 60.74 | 2.83 | 0.193 |  |  |  |
|  | Double Support (%GC) | 20.34 | 2.63 | 21.68 | 3.47 | 21.49 | 5.22 | 0.208 |  |  |  |
|  | Single Support (%GC) | 39.53 | 1.68 | 38.64 | 1.97 | 39.25 | 3.10 | 0.143 |  |  |  |

Note: ST = spatiotemporal, SD = standard deviation, %GC = % Gait Cycle, 0.000 = 0.0001

| **Supplementary data Table S3:** Two-step cluster analysis comparing DHI with spatiotemporal gait parameters for HS, UV, and BV participants. Two clusters were obtained and the distribution of patients across clusters is illustrated, delineating the corresponding proportions of individuals in the HS, UV, and HS cohorts. | | | | | | | |
| --- | --- | --- | --- | --- | --- | --- | --- |
|  |  | **Groups** | | | | | |
|  |  | **HS** | | **UV** | | **BV** | |
|  |  | Cluster 1 (%) | Cluster 2 (%) | Cluster 1 (%) | Cluster 2 (%) | Cluster 1 (%) | Cluster 2 (%) |
| **Self selected speed** | **Parameter ST** |  | | | | | |
| Slow | | | | | | | |
|  | Walking Speed (D) | 70.0% | 30.0% | 10.0% | 90.0% | 22.2% | 77.8% |
|  | Cadence (D) | 100.0% | 0.0% | 60.0% | 40.0% | 22.2% | 77.8% |
|  | Step Length (D) | 0.0% | 100.0% | 40.0% | 60.0% | 66.7% | 33.3% |
|  | Step Width (D) | 11.1% | 88.9% | 50.0% | 50.0% | 77.8% | 22.2% |
|  | Foot Off | 0.0% | 100.0% | 30.0% | 70.0% | 66.7% | 33.3% |
|  | Single Support | 0.0% | 100.0% | 30.0% | 70.0% | 66.7% | 33.3% |
|  | Double Support | 0.0% | 100.0% | 30.0% | 70.0% | 66.7% | 33.3% |
| Comfortable | | | | | | | |
|  | Walking Speed (D) | 100.0% | 0.0% | 30.0% | 70.0% | 22.2% | 77.8% |
|  | Cadence (D) | 0.0% | 100.0% | 30.0% | 70.0% | 66.7% | 33.3% |
|  | Step Length (D) | 0.0% | 100.0% | 40.0% | 60.0% | 66.7% | 33.3% |
|  | Step Width (D) | 0.0% | 100.0% | 40.0% | 60.0% | 44.4% | 55.6% |
|  | Foot Off | 0.0% | 100.0% | 40.0% | 60.0% | 55.6% | 44.4% |
|  | Single Support | 100.0% | 0.0% | 40.0% | 60.0% | 66.7% | 33.3% |
|  | Double Support | 0.0% | 100.0% | 30.0% | 70.0% | 66.7% | 33.3% |
| Fast | | | | | | | |
|  | Walking Speed (D) | 90.0% | 10.0% | 40.0% | 60.0% | 33.3% | 66.7% |
|  | Cadence (D) | 0.0% | 100.0% | 30.0% | 70.0% | 66.7% | 33.3% |
|  | Step Length (D) | 100.0% | 0.0% | 70.0% | 30.0% | 33.3% | 66.7% |
|  | Step Width (D) | 100.0% | 0.0% | 70.0% | 30.0% | 44.4% | 55.6% |
|  | Foot Off | 0.0% | 100.0% | 30.0% | 70.0% | 55.6% | 44.4% |
|  | Single Support | 100.0% | 0.0% | 70.0% | 30.0% | 44.4% | 55.6% |
|  | Double Support | 0.0% | 100.0% | 30.0% | 70.0% | 66.7% | 33.3% |
